# Ramadan Fasting: Perception and maternal outcomes during Pregnancy

**DOI:** 10.12669/pjms.37.5.4109

**Published:** 2021

**Authors:** Nazli Hossain, Mahwish Samuel, Saba Mughal, Kashif Shafique

**Affiliations:** 1 Nazli Hossain, FCPS, MBE. Department of Obstetrics & Gynecology Unit II, Dow Medical College & Ruth Pfau KM Civil Hospital, Karachi, Pakistan; 2 Mahwish Samuel, MBBS. Resident Trainee, Holy Family Hospital, Karachi, Pakistan; 3 Saba Mughal, M.Phil. School of Public Health, Dow University of Health Sciences, Karachi, Pakistan; 4 Kashif Shafique, MBBS, MPH, PhD. School of Public Health, Dow University of Health Sciences, Karachi, Pakistan

**Keywords:** Ramadan, Fasting, Preterm delivery, Gestational diabetes mellitus

## Abstract

**Objectives:**

To see perception and knowledge of women about Ramadan fasting and maternal effects of fasting.

**Methods:**

The study design was prospective, case-controlled. This study was conducted at Holy Family Hospital from 1^st^ May 2020 to July 2020. Pregnant women with spontaneous conception and singleton pregnancies, who fasted for seven or more days, were cases, and those who did not fast were taken as controls. Questionnaire was filled regarding perception of women about maternal fasting. Primary maternal outcomes included preterm delivery, pregnancy induced hypertension, and gestational diabetes mellitus. The analysis was conducted using Statistical Package for Social Sciences version 16.0.

**Results:**

A total of 215 women were included in the study, 123 women fasted, and 92 women did not fast. Only 2.8% of women knew that fasting is forbidden in pregnancy. Sixty five percent of women reported weakness as the main reason for not fasting. The rate of gestational diabetes, pregnancy induced hypertension and preterm delivery was higher among women who fasted (17% vs 14%, 7% vs 2%, 9% vs 9%) respectively, compared to non-fasting women, but were not found statistically significant. There was no difference in anthropometric measurements of newborn, among both groups.

**Conclusion:**

Ramadan fasting does not affect maternal outcomes during pregnancy.

## INTRODUCTION

Ramadan is the ninth month in the lunar calendar. During this month Muslims fast from sunrise to sunset. They are required to abstain from eating, drinking and sexual act during this time period. The duration varies according to the geographical location. The duration in the city of Karachi, Pakistan for last few years has varied between 14-15 hours. During the study period Ramadan started on April 25, 2020 with temperatures varying between 27- 37 degree Celsius.

There are around two billion Muslims in the world. Fasting is obligatory for Muslims, and is considered amongst one of the five pillars of Islam, by all schools of thought. There is relaxation for elderly and sick people, for those who are travelling and also for pregnant, breast-feeding and menstruating women.^1^ The women are required to replace or expiate the lost days, at a later date. Though the verdict is clear, majority of Muslim women continue to fast, while being pregnant. Studies have been done to find out reason for fasting, while being pregnant. Common reasons which have been identified include pregnant women’s perception that it is obligatory for them also during pregnancy, socially more acceptable, fear that they may not be able to cover the days lost.^2^ There is confluence of social, cultural and spiritual factors that drives women to fast while being pregnant. The views of physicians are also controversial on the subject. All the pregnant women seek advice from their caretakers whether they can fast or not. An internet based survey regarding physicians perception of maternal fasting found physicians advising against it during second, third trimester, whereas fasting during first trimester was considered controversial.^3^ Guidelines have also been issued for pregnant women who intend to fast during Ramadan, by various governing bodies.^4^

Though there have been studies on maternal outcome for fasting during Ramadan, there is no consensus on different aspects. A number of aspects among pregnant women have been evaluated including amniotic fluid index, fetal breathing movements, lipid and blood sugar profiles, and maternal weight gain.^5^-^7^ Investigators have also looked at the long-term outcomes of newborns to pregnant fasting mothers. Our group has previously reported upon the perinatal outcome among fasting and non-fasting women.^8^ The results showed that fasting during pregnancy did affect birth weight, especially in boys, and also affected the placental weight at the time of delivery.

Sakar et al in a prospective case control of 150 women, found a significant increase in the amniotic fluid index in non-fasting women, compared to fasting pregnant women.^9^ This decrease in amniotic fluid index has also been observed by other investigators, though they did not find any changes in the fetal biometry among pregnant fasting and non-fasting women.^5^

Fasting during the month of Ramadan has also been evaluated for preterm delivery. In a prospective cohort of around 400 pregnant women Awwad J et al did not find any difference in preterm delivery among fasting and non-fasting women.^10^ Preterm delivery was also not affected by gestational age. In a study of Iraqi women, maternal fasting was associated with decreased prevalence of gestational diabetes, when compared with non-fasting mothers.^11^

The aim of this prospective study was to determine maternal perception and outcome of fasting during the month of Ramadan.

## METHODS

This prospective case-control study was carried out at Holy Family Hospital, Karachi. The study period was from 1^st^ May 2020, to July 2020. It is a secondary care hospital, which caters to the lower middle class income group. It has annual delivery rate of around 2500 deliveries.

The data collection started at the end of month of Ramadan. Pregnant women were divided in two groups, those who fasted and those who did not fast during the month of Ramadan. Those who fasted for more than seven days, either regularly or randomly were included among the fasting group.

### Inclusion criteria

It included singleton pregnancy, spontaneous conception.

### Exclusion criteria

It included multiple pregnancies, women with known medical complications like essential diabetes mellitus, chronic hypertension, intrauterine growth restriction, intrauterine demise. Also women who conceived with assisted conception were excluded.

The maternal outcomes included preterm delivery, gestational diabetes mellitus, pregnancy induced hypertension. The perinatal outcome included birth weight and anthropometric measurements of newborn.

Gestational age was calculated from the last menstrual period or viability scan, which ever was available. Preterm delivery was defined as birth before 37 weeks of gestation. Also body mass index was calculated at the beginning of pregnancy and increase in maternal weight was recorded, at the end of pregnancy.Pregnancy induced hypertension was defined as onset of new hypertension after 20 weeks of pregnancy, with systolic blood pressure of ≥ 140 mm hg, and diastolic blood pressure of ≥ 80 mm hg, with or without proteinuria.^12^

Gestational diabetes mellitus, was identified with the two hour oral glucose tolerance test, routinely done at the institute between 24-28 weeks of gestation.^13^ Diagnosis of gestational diabetes mellitus was made if one or more of blood glucose levels were raised after 75gm glucose load. Also mode of delivery was noted among both groups of women. Baby’s birth weight, height and length were also noted between both groups of women.

All the above details were filled in a questionnaire, after a face-to-face interview by one of the investigators. The questionnaire had three parts. First part included demographic details, second part included perception of women about maternal fasting, along with side effects of fasting, they experienced. Third part included details about maternal and perinatal outcomes.

The permission was taken from the Ethical review board of Holy Family hospital to carry out the research study (HFH/ 296/2020, 14-4-2020). Women were approached following delivery, after informed verbal consent.

### Statistical Analysis

The analysis was conducted using Statistical Package for Social Sciences version 16.0. Descriptive statistics were reported as percentage and frequency for categorical variables, whereas mean and standard deviation were reported for quantitative variables. Chi-square test was used to determine the relationship between the maternal outcomes and fasting status during pregnancy. Assumption of normality was checked for quantitative variables by Shapiro Wilk test and Mann-Whitney test was used to determine mean differences for perinatal outcomes and blood profile between fasting and non-fasting pregnant women. All test results having p-values less than or equal to alpha (0.05) were considered statistically significant.

## RESULTS

A total of 215 pregnant women were included in this study. Women who reported that there is a need to fast at later date were 39.5% (n=85) whereas 34.4% (n=74) women considered fasting during pregnancy is essential. Only 2.8% (n=6) reported that fasting is forbidden during pregnancy. Among all 57.2% (n=123) women fasted this year during pregnancy. Majority of the females (65.8%, n=102) reported weakness one of the reason of not fasting. (Table-I).

**Table-1:** Knowledge, perception and practice of pregnant women of fasting during Ramadan (n=215).

Characteristics	n	%
***Husband approval for fasting***		
Yes	200	93.0
No	15	7.0
***In laws approval for fasting***		
Yes	198	92.1
No	17	7.9
Knowledge*		
Essential [Wajib]	74	34.4
Recommended	50	23.3
Not recommended	4	1.9
Need to fast at later date	85	39.5
Forbidden during pregnancy	6	2.8
Don’t know	16	7.4
***Fast in last 3 years during pregnancy***		
Yes	96	44.7
No	119	55.3
***Fast this year during pregnancy***		
Yes	123	57.2
No	92	42.8
***Pattern (n=123)***		
Regular	100	81.3
Random	13	10.6
Alternate/ Once a week	10	8.1
***Consult***		
Family	13	6.0
Relative	3	1.4
Doctor	16	7.4
Mufti	8	3.7
Didn’t consult	175	81.4
Reason for not fasting* (n=155)		
Fatigue	26	16.8
Weakness	102	65.8
Will harm fetus/ me	43	27.7
Not required during pregnancy	2	1.3
Vomiting	22	14.2
Prohibited by doctor	8	5.2
Medical conditions	7	4.5

	***Mean ± SD***	***Range***

***Total days of fasting***		
	12.13 ± 12.77	0 - 29

* Multiple options were selected.

Average BMI (kg/m^2^) at start of pregnancy was significantly different in women who were fasting during pregnancy (25.44 ± 5.31) and those who were not fasting (23.97 ± 4.69) (p-value=0.036). It was noted that the proportion of gestational diabetes was high among fasted women (17.1%, n=21) as compared to those who did not fast (14.1%, n=13) during pregnancy and It was also observed that women who fasted were more prone to pregnancy induced hypertension (5.7%, n=7) as compared to those who did not fast (2.2%, n=2) however gestational diabetes and hypertension were not significantly associated with the fasting. Those women who did not fast were more likely to have preterm delivery (9.8%, n=9) as compared to those who fasted (7.3%, n=9) but the association was not found to be significant. Tendency of having spontaneous vaginal delivery was high in fasted women (39.0%, n=48) as compared to those who did not fast (29.3%, n=27) (p-value=0.049) (Table-II).

**Table-II:** Sociodemographic characteristics and maternal outcomes by fasting during Ramadan (n=215).

Variables	Fasted	Not fasted	p-value
	***(n = 123)***	***(n = 92)***	
***Age in years, (Mean ± SD)***	26.54 ± 4.82	26.84 ± 5.06	0.955*
***Gestational age in weeks***	38.06 ± 1.20	37.59 ± 1.36	0.008*
***BMI at start of pregnancy in kg/m2***	25.44 ± 5.31	23.97 ± 4.69	0.036*
***Weight gain during pregnancy in kg***	7.68 ± 4.87	8.11 ± 3.99	0.234*
***Education, n (%)***			
Illiterate	2 (1.6)	3 (3.3)	0.216†
Secondary	9 (7.3)	14 (15.2)	
Matric	32 (26.0)	22 (23.9)	
Intermediate	51 (41.5)	28 (30.4)	
Graduate/ Masters	29 (23.6)	25 (27.2)	
***Monthly income in PKR, n (%)***			
10 - 15	15 (12.2)	5 (5.4)	0.315†
15 - 20	38 (30.9)	35 (38.0)	
20 - 50	56 (45.5)	40 (43.5)	
> 50	14 (11.4)	12 (13.0)	
***Parity***			
0	36 (29.3)	30 (32.6)	0.771†
1	29 (23.6)	24 (26.1)	
2	36 (29.3)	26 (28.3)	
3 or more	22 (17.9)	12 (13.0)	
***GDM***			
Yes	21 (17.1)	13 (14.1)	0.558†
No	102 (82.9)	79 (85.9)	
***PTD***			
Yes	9 (7.3)	9 (9.8)	0.518†
No	114 (92.7)	83 (90.2)	
***PIH***			
Yes	7 (5.7)	2 (2.2)	0.306†
No	116 (94.3)	90 (97.8)	
***Mode***			
SVD/Instruments	48 (39.0)	27 (29.3)	0.049†
LSCS	73 (59.3)	58 (63.0)	
Emergency LSCS	2 (1.6)	7 (7.6)	

BMI=Body Mass Index, GDM=Gestational diabetes mellitus, PTD=Pre-Term Delivery,

PIH=Pregnancy-Induced Hypertension, SVD=Spontaneous Vaginal Delivery,

LSCS=Lower Segment Cesarean Section.

* p-value calculated by Mann-Whitney U test.

† p-value calculated by Chi-square test. † p-value calculated by Fisher’s exact test.

It was observed that perinatal outcomes did not show any statistically significant mean differences between the groups of fasted and non-fasted pregnant women. Table-III. However, 5-minute Apgar score of new born was slightly higher in fasted women (9.00 ± 0.01 as compared to those who did not fast (8.92 ± 0.53) (p-value=0.044).

**Table-III:** Perinatal outcome by fasting and non-fasting pregnant women (n=215).

Variables	Fasted (n = 123)	Not fasted (n = 92)	p-value
Birth weight in kg, (Mean ± SD)	2.87 ± 0.39	2.83 ± 0.43	0.301*
Height of baby in cm	50.74 ± 3.16	50.16 ± 3.46	0.222*
head circumference in cm	33.96 ± 1.42	34.09 ± 1.96	0.808*
Apgar score at 1-min	7.99 ± 0.09	7.92 ± 0.63	0.398*
Apgar score at 5-min	9.00 ± 0.01	8.92 ± 0.53	0.044*
***Sex of baby, n (%)***			
Male	66 (53.7)	48 (52.2)	0.829†
Female	57 (46.3)	44 (47.8)	

* p-value calculated by Mann-Whitney U test.

† p-value calculated by Chi-square test.

Complete blood profile was reported before Ramadan and after delivery. Average blood profile was not found to be significantly different among fasted and non-fasted women before Ramadan and similar results were observed even after delivery. (Table-IV)

**Table-IV:** Complete blood profile of fasted and non-fasted pregnant women (n=215).

Variables	Fasted (n = 123)	Not fasted (n = 92)	p-value*
***Blood profile before Ramadan***			
Hemoglobin	11.10 ± 1.12	11.13 ± 1.21	0.744
Hct	33.60 ± 2.82	33.63 ± 3.03	0.914
Neutrophils	72.01 ± 5.85	70.92 ± 5.83	0.070
Platelets	277.98 ± 76.07	267.97 ± 73.45	0.391
***Blood profile after delivery***			
Hemoglobin	10.47 ± 1.23	10. 48 ± 1.30	0.914
Hct	32.09 ± 3.34	31.96 ± 3.40	0.681
Neutrophils	74.62 ± 6.71	74.10 ± 7.65	0.585
Platelets	234.95 ± 80.90	229.52 ± 84.47	0.583

(Mean ± SD) are reported.

* p-value calculated by Mann-Whitney U test.

## DISCUSSION

The Islamic rulings for exemption from fasting during the month of Ramadan for menstruating, breast feeding and pregnant women are very clear. Yet, a number of women continue to observe fasting during the holy month. Though 57% of the women in the study fasted, but only few 2.8% had correct knowledge of Islamic rulings about it. Majority of the women in the study were educated, and 80% of them did not consider any need to consult a religious scholar for Islamic rulings on fasting during pregnancy. Only 7.4% consulted their doctors before fasting. More than 70% of the women found fatigue and weakness as the main barrier in fasting. Only 27% thought, it’s going to be harm their fetus.

Change in diet and eating pattern are commonly observed during the month of Ramadan. In our study, both groups of women showed nearly identical weight gain during pregnancy. The body mass index of women who fasted was comparatively more than non-fasting mothers 25 vs 23 kg/m^2^. A number of aspects of maternal fasting on pregnancy have been explored. Increased frequency of hyperemesis gravidarum, improved metabolic profile, changes in the amniotic fluid index, birth weight of newborn.

The rate of preterm deliveries was not significantly affected by the maternal fasting, in our study. In a recent study from Pakistan, preterm delivery was seen in 6.9% of fasting women, compared to 5.49% in non-fasting women.^14^ Other investigators have also made similar observations. In a prospective cohort of more than 400 women, duration of gestation and birth weight was not found to be affected by maternal fasting. Preterm delivery was not found to be associated with gestational age at fasting.^10^ The risk of very preterm, defined as delivery between 28-31 weeks of gestation has been found to be increased in a population based cohort from Canada. The study utilized the data from birth certificates of Arabic women giving birth in Quebec region of Canada. The background rate of preterm delivery in the Arabic population, residing in Quebec was 5.53 per 100 births.^15^

The number of women who developed pregnancy induced hypertension, in the study was more in the fasting group, though it was not found statistically significant. This may be attributed to decrease in serum calcium level during fasting. A decreased serum calcium level has been found to be associated with hypertension during pregnancy.^16^ We did not measure serum calcium levels, in both groups of women.

We did not find any significant differences in anthropometric measurements of newborn, in both groups of women. The average birth weight of babies in both groups was nearly identical. Similar observations were made in a prospective study of 400 women, who were matched according to their trimesters, fasting during Ramadan, did not affect fetal outcome.^17^ This was also in line with our previous research on maternal fasting and perinatal outcome.^8^ We did not find any changes in the hematological parameters of women before and after the fasting month. This also helps in counseling women when they complain about weakness and fatigue following fasting month.

Though, our study is facility based, and has a limited sample size, it does provide guidelines to physicians that maternal fasting is not harmful for mothers and the baby. Evaluation of amniotic fluid index among both groups of women would have strengthened the study, but was not done due to financial constraints.

Pakistan being a Muslim country, where majority of people practice fasting during the month of Ramadan. Women constitute more than half of the population. There is dearth of studies focusing on maternal and perinatal aspects of Ramadan fasting. There is need to plan large population based studies, to have a consensus opinion on how Ramadan fasting affects maternal and perinatal outcome. These can be conducted on country wise basis, among different institutes. This will help health care professionals in providing clear guidelines to pregnant women about the risks of fasting. Similarly, there is need to plan follow up studies in the newborns, to know the long term effects of maternal fasting.

### Author’s Contribution:

**NH:** Conceived the idea, and did write up. Responsible for integrity of submitted work.

**MS:** Collected data and followed patient.

**SM & KS:** Did data analysis and contributed in write up.

## References

[1] The Holy Quran Surah 2, Verse 185.

[2] Mubeen SM, Mansoor S, Hussain A, Qadir S (2012). Perceptions and practices of fasting in Ramadan during pregnancy in Pakistan. Iran J Nurs Midwifery Res.

[3] Abd-El-Aal DE, Shahin AY, Hamed HO (2009). Effect of short-term maternal fasting in the third trimester on uterine, umbilical, and fetal middle cerebral artery Doppler indices. Int J Gynaecol Obstet.

[4] Bajaj S, Khan A, Fathima FN, Jaleel MA, Sheikh A, Azad K (2012). South Asian consensus statement on women's health and Ramadan. Indian J Endocrinol Metabol.

[5] Seckin KD, Yeral MI, Karsli MF, Gultekin IB (2014). Effect of maternal fasting for religious beliefs on fetal sonographic findings and neonatal outcomes. Int J Gynaecol Obstet.

[6] Ziaee V, Kihanidoost Z, Younesian M, Akhavirad MB, Bateni F, Kazemianfar Z (2010). The effect of ramadan fasting on outcome of pregnancy. Iranian J Pediatr.

[7] Ziaee V, Razaei M, Ahmadinejad Z, Shaikh H, Yousefi R, Yarmohammadi L (2006). The changes of metabolic profile and weight during Ramadan fasting. Singapore Med J.

[8] Gul Z, Rajar S, Shaikh ZF, Shafique K, Hossain N (2018). Perinatal outcome among fasting and non fasting mothers during the month of Ramadan. Pak J Med Sci.

[9] Sakar MN, Gultekin H, Demir B, Bakir VL, Balsak D, Vuruskan E (2015). Ramadan fasting and pregnancy:implications for fetal development in summer season. J Perinatal Med.

[10] Awwad J, Usta IM, Succar J, Musallam KM, Ghazeeri G, Nassar AH (2012). The effect of maternal fasting during Ramadan on preterm delivery:A Prospective Cohort Study. BJOG:Int J Obstet Gynaecol.

[11] Safari K, Piro TJ, Ahmad HM (2019). Perspectives and pregnancy outcomes of maternal Ramadan fasting in the second trimester of pregnancy. BMC Pregnancy Childbirth.

[12] Tranquilli AL, Dekker G, Magee L, Roberts J, Sibai BM, Steyn W (2014). The classification, diagnosis and management of the hypertensive disorders of pregnancy:A revised statement from the ISSHP. Pregnancy Hypertension.

[13] Diagnostic criteria and classification of hyperglycaemia first detected in pregnancy:a World Health Organization Guideline (2014). Diabetes Res Clin Pract.

[14] Parveen R, Khakwani M, Latif M, Tareen AU (2020). Maternal and Perinatal outcome after Ramadan Fasting. Pak J Med Sci.

[15] Tith RM, Bilodeau-Bertrand M, Lee GE, Healy-Profitos J, Auger N (2019). Fasting during Ramadan Increases Risk of Very Preterm Birth among Arabic-Speaking Women. J Nutrition.

[16] Almaghamsi A, Almalki MH, Buhary BM (2018). Hypocalcemia in Pregnancy:A Clinical Review Update. Oman Med J.

[17] Karateke A, Kaplanoglu M, Avci F, Kurt RK, Baloglu A (2015). The effect of Ramadan fasting on fetal development. Pak J Med Sci.

